# Foreign Body Embedded in Anterior Chamber Angle

**DOI:** 10.1155/2012/631728

**Published:** 2012-10-04

**Authors:** Shmuel Graffi, Beatrice Tiosano, Ran Ben Cnaan, Jonathan Bahir, Modi Naftali

**Affiliations:** Department of Ophthalmology, Baruch Padeh Medical Center and Faculty of Medicine, Bar Ilan University, 15208 Galilee, Israel

## Abstract

*Introduction*. We present a case of a metallic foreign body embedded in the anterior chamber angle. After standing in close proximity to a construction worker breaking a tile, a 26-year-old woman using soft contact lens for the correction of mild myopia presented to emergency department for evaluation of a foreign body sensation of her right eye. *Methods and Results*. Diagnosis was confirmed by gonioscopic examination and a noncontrast CT scan of head and orbits. The foreign body was removed by an external approach without utilizing a magnet. The patient's final outcome was favorable. *Discussion*. The above is a rare clinical situation, which is impossible to detect on slit-lamp examination without a gonioscopic view. Proper imaging and a specific management are mandatory in order to achieve favorable outcome.

## 1. Introduction

Penetrating ocular trauma is a potentially vision-threatening injury. The extent of injury depends on factors that include size and composition of the foreign body, force of entry into the eye, location of the resulting wound, and the final location of the foreign body. Other important factors that might influence the final prognosis include initial visual acuity, presence of an afferent pupillary defect, perforation of the globe, and endophthalmitis. Early diagnosis and removal of the foreign body are essential for favorable outcome. In some cases, the area of penetration and the intraocular foreign body (IOFB) itself may not be easily detected. One such example is the presence of an IOFB lodged in the inferior chamber angle making it impossible to detect without performing gonioscopic examination.

## 2. Case Report

A 26-year-old woman using soft contact lens for the correction of mild myopia presents to emergency department for evaluation of a foreign body sensation of her right eye. On history, she reports standing in close proximity to a construction worker breaking a tile wall, when she felt a sudden onset of foreign body sensation. On initial examination, the patient's visual acuity was 6/6-OD and 6/6 OS. The intraocular pressure was 13 on the right eye and 15 on the left. A slit-lamp examination of her left eye was within normal limits. Her right eye examination was notable for mild conjunctival injection, a thin self-sealing corneal wound. This wound was initially estimated as a linear erosion of 2.5 millimeters in length located in the upper nasal quadrant and was Seidel negative. The anterior chamber depth was normal with few cells and no glair. The iris showed no disruption. The lens was clear, and examination of the posterior segment was unremarkable. Due to a high degree of clinical suspicion, a gonioscopic examination was performed showing a small dark foreign body embedded in the inferior chamber angle at 7 o'clock. An entry point was visible on the soft contact lens used at the time of injury confirming that the entrance wound was at the cornea.

**Figure 1:**
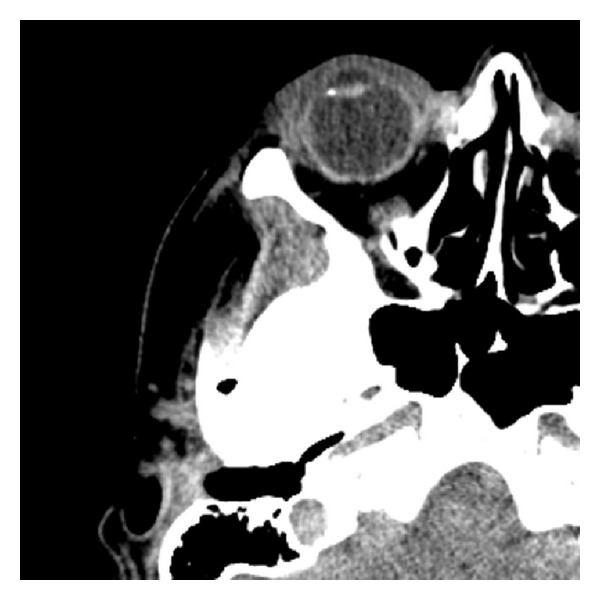
A noncontrast CT scan demonstrating foreign body resting on the zonules area.

**Figure 2:**
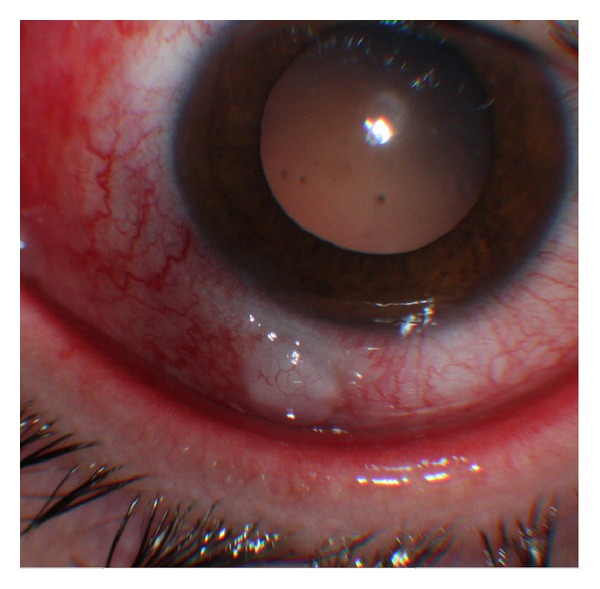
An unintentional conjunctival bleb at 7 o'clock.

**Figure 3:**
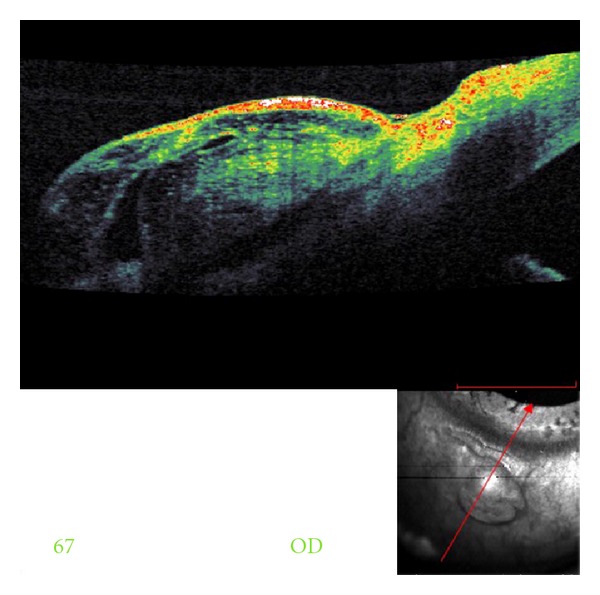
An anterior segment OCT demonstrating scleral flap covered by conjunctiva.

A noncontrast CT scan (NC-CT) of head and orbits revealed a regular border radiopaque foreign body in the chamber angle, anterior to the lens. The patient was taken for surgical intervention. Removal of the foreign body via limbal incision entering on the superior aspect was complicated by disappearance of the object from the surgeons view. The procedure was stopped, and the patient was taken immediately for another NC-CT with concern for passage of the IOFB into the posterior segment. The CT demonstrated the IOFB resting in the zonules area adjacent to the lens (Figure 1). 24 hours later using a slit lamp with gonioscopy, the location of the IOFB was marked by an intentional corneal abrasion followed by a fluorescein staining. A bilevel wide opening above the marking exposed the foreign body, allowing for its uncomplicated removal. The IOFB was identified by a forensic lab as an iron. Two months following surgery, an unintentional bleb was observed (Figure 2). Anterior segment optical coherence tomography (ASOCT) showed a scleral flap covered by conjunctiva (Figure 3). The patient reported that she was unable to wear her contact lens, likely secondary to this finding. Despite normal intraocular pressure, a surgery for resecting the bleb was scheduled. However, surgery was canceled due to acute conjunctivitis. Six months later, the patient reported improvement, and examination revealed a significant flattening of the bleb, which allows for contact lens use. Of note, the patient's visual acuity was 6/6 OU.

## 3. Discussion

A few cases of intraocular foreign bodies presenting in the anterior chamber angle have been reported. Our patient had a metallic foreign body entering through the cornea and resting in the inferior chamber angle making it impossible to detect on slit-lamp examination without a gonioscopic view. Therefore, after taking a thorough history of any ocular or orbital trauma, foreign body must be ruled out even when not observed on initial examination. Gonioscopy is recommended only if there is no risk of extrusion of intraocular content [1]. Thereafter, a proper imaging study should be performed in order to confirm the diagnosis and rule out additional foreign bodies. While magnetic resonance imaging is contraindicated when dealing with a metallic foreign body, CT scan is considered the gold standard. Ultrasound biomicroscopy and ASOCT may further add to the diagnosis by estimating more accurately the foreign body size and location [2, 3]. The two main surgical approaches for removing metallic IOFBs are the external approach using a large electromagnet and the internal approach which exhibits vitrectomy followed by forceps or internal magnet use [4]. In this case, the first surgical attempt to remove the foreign body approaching from the superior aspect through a limbal incision failed. The second attempt, performed by a glaucoma specialist, utilized the external approach for removing the foreign body. Both surgeons did not use an intraoperative magnet because the IOFB was suspected to be a piece of ceramic tile. This decision was based on the patient's history and the lack of artifact rays on CT. Many patients wish to use contact lens as soon as possible. The resolution of this bleb without surgical intervention may support the use of “watch and wait” period of few months, after which reevaluation of the injury may reduce the number of operations.

Our case demonstrates the need for a thorough history and physical examination when examining a patient who sustains ocular trauma. In addition, proper diagnostic tests including gonioscopy should be added for a proper management of the patient.
